# Real-world outcomes and prognostic factors in anaplastic thyroid cancer: evidence from the REGETNE-Thyroid cohort

**DOI:** 10.1093/oncolo/oyag158

**Published:** 2026-05-21

**Authors:** Pau Mascaró-Baselga, José Miguel Rodellar Sanz, Tiago Nunes da Silva, Jorge Hernando, Alejandro García-Álvarez, Carlos González Merino, Javier Molina-Cerrillo, Antia Fernandez-Pombo, German Iglesias-Alvarez, Isaac Ceballos Lenza, María Plana Serrahima, Maria Rosa Bella Cueto, Maria Luisa Isidro, Javier Martinez Trufero, Gloria Marquina, Nieves Martinez, Guillermo Crespo, Raquel Jimeno, Isabel Lorenzo, Pablo Ayala de Miguel, Victoria Alcazar, Sandra Martínez-Badal, Jaume Capdevila, Teresa Alonso-Gordoa

**Affiliations:** Vall Hebron Institute of Oncology, Barcelona, Spain; Hospital Universitario Ramón y Cajal, IRYCIS, Madrid, Spain; Instituto Português de Oncologia de Lisboa Francisco Gentil, Lisbon, Portugal; Vall Hebron Institute of Oncology, Barcelona, Spain; Vall Hebron Institute of Oncology, Barcelona, Spain; Hospital Universitario Ramón y Cajal, IRYCIS, Madrid, Spain; Hospital Universitario Ramón y Cajal, IRYCIS, Madrid, Spain; University Hospital Complex of Santiago, Santiago de Compostela, Spain; Central University Hospital of Asturias, Oviedo, Spain; University Hospital of Canarias, San Cristobal de La Laguna, Spain; Catalan Intitute of Oncology L’Hospitalet, L’Hospitalet de Llobregat, Barcelona, Spain; Parc Taulí Hospital Universitari. Institut d‘Investigació i Innovació Parc Taulí (I3PT-CERCA). Universitat Autònoma de Barcelona, Sabadell, Spain; University Hospital of A Coruña, A Coruña, Spain; Miguel Servet University Hospital, Zaragoza, Spain; Hospital Clínico San Carlos, Department of Medicine, School of Medicine, Universidad Complutense de Madrid (UCM), Instituto de Investigación Sanitaria (IdISSC), EURACAN Referral Centre, Madrid, Spain; University Hospital Complex of Santiago, Santiago de Compostela, Spain; Burgos University Hospital, Burgos, Spain; Marqués de Valdecilla University Hospital, Santander, Spain; University Hospital Complex of Vigo, Vigo, Spain; Hospital Complex of Cáceres, Cáceres, Spain; Severo Ochoa University Hospital, Madrid, Spain; Vall Hebron Institute of Oncology, Barcelona, Spain; Vall Hebron Institute of Oncology, Barcelona, Spain; Hospital Universitario Ramón y Cajal, IRYCIS, Madrid, Spain

**Keywords:** Thyroid carcinoma, anaplastic, molecular diagnostic techniques, molecular targeted therapy, immunotherapy, tyrosine kinase inhibitors

## Abstract

**Background:**

Anaplastic thyroid cancer (ATC) is an extremely rare cancer with very poor prognosis. Targeted therapies have improved outcomes in selected patients, but molecular testing is often difficult because of the aggressive course, rapid clinical deterioration, and long turnaround times. This study aimed to provide real-world evidence on the management and outcomes of ATC and to evaluate the prognostic impact of molecular testing.

**Materials and methods:**

This multicentric, retrospective study included patients diagnosed with ATC between 1992 and 2024 from the REGETNE-Thyroid Network. Factors associated with overall survival (OS) were analyzed using Cox proportional hazards models.

**Results:**

214 patients were included (median age 70.8 years; 56.5% female). Median OS was 4.5 months. Access to molecular testing (HR 0.42, *p = *0.001) and *BRAF V600E* mutation (HR 0.50, *p = *0.008) were associated with longer OS, whereas *TERT* promoter mutations were associated with shorter survival (HR 2.80, *p = *0.014). Local surgery showed a favorable prognostic association, regardless of stage (IVa/IVb: HR 0.54, *p = *0.026; IVc: HR 0.36, *p <* 0.001). In metastatic disease, immunotherapy (HR 0.31; *p <* 0.001), tyrosine kinase inhibitors (HR 0.40; *p = *0.002) and their combination (HR 0.24; *p = *0.001) significantly improved OS, whereas chemotherapy did not (HR 0.76; *p = *0.096). Targeted therapy, used predominantly (>95%) in *BRAF V600E-*mutated ATC, was also associated with longer survival (HR 0.40; *p = *0.001).

**Conclusion:**

Molecular testing was associated with improved survival in ATC, likely driven by the identification of actionable alterations with effective targeted therapies. Local management is key in all stages. Our findings also support immunotherapy, tyrosine kinase inhibitors and their combinations as key therapeutic options.

Implications for Practice Management of anaplastic thyroid cancer should incorporate early molecular testing whenever feasible, as access to molecular profiling is associated with improved survival and enables use of effective targeted therapies. In routine practice, immunotherapy and tyrosine kinase inhibitors—alone or in combination—provide clinically meaningful benefit, whereas chemotherapy offers limited survival impact. These data support a treatment paradigm centered on rapid molecular characterization, modern systemic therapies, and proactive local disease control to improve outcomes in this rapidly progressive malignancy.

## Introduction

Anaplastic thyroid cancer (ATC) is a rare but highly aggressive thyroid malignancy, accounting for 1–2% of all thyroid cancers.^1^ Despite its low incidence, ATC carries a dismal prognosis, with a median overall survival (OS) ranging from 4 to 6 months and 1-year OS below 20%.^2^ Approximately 70–80% of patients present with locally advanced or metastatic disease and experience rapid clinical deterioration.

Molecular profiling of ATC has revealed frequent genetic alterations, the most common being *TERT* promoter mutations (73%), followed by *TP53* (65%), *BRAF* (45%), *NRAS* (22%), and PIK3CA (18%).^3–6^ In a subset of cases, ATC may arise through dedifferentiation from differentiated thyroid carcinoma (DTC) or poorly differentiated thyroid carcinoma (PDTC), often involving early alterations in the MAPK pathway (*BRAF* or *RAS*) and subsequent acquisition of mutations in *TERT*, *TP53*, or *PI3K*-*AKT* signaling-related genes.^7^^,^^8^

Advanced ATC has poor response to conventional chemotherapy (taxanes, anthracyclines, platinum agents).^3^ However, in recent years targeted therapies (TTs) have shown substantial benefit in selected subgroups. In this context, the phase II ROAR basket trial,^9^ which evaluated the combination of dabrafenib and trametinib in *BRAF* V600E–mutant cancers, reported an objective response rate (ORR) of 56% in the ATC cohort, with a median progression-free survival (PFS) of 6.7 months, and a median OS of 14.7 months.^10^ Other less frequent alterations in ATC, such as *NTRK* fusions (3%), *RET* alterations (<1%) and *ALK* rearrangements (<1%), are also actionable and thus, therapeutically relevant. In the LIBRETTO-001 basket study, patients with previously treated *RET* fusion-positive thyroid cancers (*n = *19, all histologic types) treated with selpercatinib achieved an ORR of 79%, including durable responses in the 2 ATC cases included in the study.^11^ Similarly, pooled analysis including data from 3 studies of *NTRK* fusion–positive thyroid cancers treated with larotrectinib^12–14^ reported an ORR of 29% in the ATC subgroup, with a median OS of 8.8 months.^15^

Given the potential benefits of molecularly guided therapies, early genomic testing is essential in patients with ATC. However, the aggressive nature of the disease and early clinical deterioration often limit the feasibility of comprehensive molecular profiling due to its longer turnaround time. In this context, rapid diagnostic techniques such as immunohistochemistry or PCR-based assays for *BRAF* mutations can be valuable in guiding early therapeutic decisions.^16–18^

Beyond TTs, immunotherapy (IO) with immune checkpoint inhibitors (ICIs) has also shown promising results in ATC (Supplemental Table 1). In a phase II trial, the anti-PD-1 spartalizumab showed an ORR of 19% and 35% in patients with PD-L1 ≥ 50%, with durable responses in the PD-L1 positive subgroup of patients.^19^ Similarly, the DUTHY study combining durvalumab and tremelimumab reported an ORR of 33% and a median OS of 13.8 months in the ATC cohort, with increased ORR (50%) among PD-L1–positive tumours.^20^Despite the limited efficacy of tyrosine kinase inhibitors (TKIs) in monotherapy in ATC,^21^ combinations of ICIs and TKIs have yielded encouraging results. In the phase II ATLEP study, the combination of lenvatinib and pembrolizumab achieved an ORR of 52%, with a median PFS and OS of 9.5 and 10.3 months in patients with ATC, respectively.^22^ Furthermore, the phase II CABATEN study investigating cabozantinib with atezolizumab combination in endocrine tumors has also shown promising results in the ATC cohort (ORR of 21.4% and median PFS of 8.4 months).^23^

Overall, ATC is a highly aggressive malignancy with limited curative options and poor prognosis. Given the rarity and aggressiveness of ATC, robust prospective data are lacking, and access to molecular testing and therapies such as TTs, IO, and TKIs remains highly variable in routine practice. This study aims to provide real-world evidence from the REGETNE-Thyroid collaborative network, to examine treatment outcomes in ATC and to evaluate the prognostic impact of molecular testing and access to novel therapies.

## Materials and methods

### Patient selection and data collection

The REGETNE-Thyroid Registry is a collaborative network coordinated by GETNE (Spanish Taskforce Group of Neuroendocrine and Endocrine Tumors), comprising 33 hospitals across Spain and Portugal with expertise in thyroid cancer management. The registry was established in 2020 and includes patients with thyroid cancer (including all histological types).

Patients diagnosed with locally advanced or metastatic ATC between March 1992 and July 2024 were identified from the REGETNE-Thyroid Registry and subsequently included in this study. Patients with incomplete clinical records were excluded.

Data collected included sex and age, performance status, disease characteristics and staging, molecular information, local and systemic treatments, and survival. Molecular studies varied according to local practices, including surrogate IHC testing for some gene mutations (e.g., *BRAF* V600E or *TP53*), PCR-based sequencing of specific genes of interest or NGS with diverse gene panels according to the platforms used.

The REGETNE-Thyroid Registry complies with Spanish data protection regulations, including Regulation (EU) 2016/679 (GDPR) and Organic Law 3/2018 (LOPD-GDD). All patient data were anonymized to ensure confidentiality. The study was approved by the corresponding ethics committee and conducted in accordance with the Declaration of Helsinki.

### Statistical analysis

The data cut-off date for the analysis was December 20, 2024.

For descriptive analyses, qualitative variables are presented as percentages and frequencies, while quantitative ones are presented as medians and interquartile ranges. For survival analyses, OS was defined as the time of ATC diagnosis and death from any cause. Patients alive at time of data cut-off were censored at their last follow-up time. Time-to-event outcomes such as OS were analyzed using Kaplan–Meier estimates, with comparisons between subgroups made using the log-rank test. Statistical significance was set at *p* ≤ 0.05 for all comparisons. Cox proportional hazards models were used to estimate hazard ratios, which were expressed as 95% confidence intervals (95%CI). The statistical analyses were performed using IBM SPSS Statistics (v25).

## Results

### Baseline and molecular characteristics

A total of 214 patients with ATC were included (Table 1). Median age at diagnosis was 70.8 years, and 121 (56.5%) were female. At diagnosis, 87 patients (40.6%) presented with locoregional disease (stage IVa/IVb), while 116 (54.2%) had metastatic disease (stage IVc). The most frequent metastatic sites were lung (36.9%), non-regional lymph nodes (13.6%), and bone (6.1%). Of 87 patients with locorregional disease, 56 (64.3%) developed metastatic progression during disease course.

**Table 1:** Baseline characteristics of the cohort.

Characteristic	*N = *214
**Clinical characteristics**	
**Female sex—n (%)**	121 (56.5)
**Age—median (IQR)**	70.8 years (61.9 – 78.6)
**Year of diagnosis—n (%)**	
** <2020**	103 (48.1)
** ≥2020**	111 (51.9)
**Initial TMN Stage—n (%)**	
** IVa**	17 (7.9)
** IVb**	70 (32.7)
** IVc**	116 (54.2)
** N/A**	11 (5.1)
**Metastatic sites *(stage IVc, n = *116) – n (%)**	
** Lung—n (%)**	79 (36.9)
** Non-regional lymph nodes—n (%)**	29 (13.5)
** Bone—n (%)**	13 (6.1)
** Liver—n (%)**	6 (2.8)
** Other—n (%)**	11 (5.1)
**Molecular characteristics**	
**Patients with molecular testing available—n (%)**	148 (69.2)
**Type of molecular study—n (%)**	
** NGS**	51 (23.8)
** PCR**	63 (29.4)
** Surrogate IHC (e.g., *BRAF* V600E)**	84 (39.3)
**Most frequent mutations *(% out of patients with available data for each gene)* – n (%)**	
** *TP53* mutation *(evaluated, n = 78)***	49 (62.8)
** *TERT*p mutation *(evaluated, n = 58)***	18 (31)
** PAX8 loss by IHC *(evaluated, n = 84)***	23 (27.4)
** *RAS* mutation *(evaluated, n = 114)***	25 (21.2)
** *BRAF* mutation *(evaluated, n = 148)***	37 (20.4)
** PIK3CA mutation *(evaluated, n = 49)***	3 (6.1)
** PTEN mutation *(evaluated, n = 51)***	3 (5.9)
** *ALK* fusion *(evaluated, n = 51)***	1 (2)
** *NTRK* fusion *(evaluated, n = 51)***	1 (2)
** *RET* fusion *(evaluated, n = 51)***	0 (0)

IHC: immunohistochemistry; IQR: interquartilic range; NGS: next generation sequencing; PCR: polymerase chain reaction; TERTp: TERT promoter.

Regarding molecular profiling, 148 patients (69.2%) underwent molecular testing (Table 1). Specifically, 84 (39.3%) had surrogate IHC analysis; 63 (29.4%) with PCR, and 51 (23.8%) with NGS. The most frequent molecular alterations were *TP53* mutations (62.8%), *TERT*p mutations (35.3%), and *BRAF* mutations (20.4%, all *BRAF* V600E). *RAS* mutations (*KRAS*, *NRAS* or *NRAS*) were identified in 21.2% of patients. An *ALK* rearrangement and an *NTRK* fusion were found in 1 patient each; no *RET* fusions were identified.

### Locorregional management of ATC

Overall, 70 patients (32.7%) underwent surgery and 36 (16.8%) had chemoradiotherapy (CRT) for local disease control, regardless of stage. Among patients with locoregional disease (stage IVa/IVb; *n = *87; Table 2), 34 (39.1%) underwent surgery, achieving a R0 resection in 16 (47.1%) patients. Additionally, 23 patients (26.4%) received CRT, predominantly administered concurrently and as a trimodal treatment with surgery. In the metastatic subgroup (stage IVc at diagnosis; *n = *116), 30 patients (25.8%) underwent surgical resection of the primary tumor and 13 (11.2%) received radiotherapy for local control.

**Table 2:** Local treatment in stage IVa/IVb disease.

Characteristic	*N = *87
***Surgery—*n *(%)***	34 (39.1)
***Type of surgery* (% of operated patients, n = 34) *–* n *(%)***	
*** Total thyroidectomy***	28 (82.4)
*** Hemithyroidectomy***	3 (8.8)
*** N/A***	3 (8.8)
***Resection margins* (% of operated patients, n = 34) *–* n *(%)***	
*** R0***	16 (47.1)
*** R1***	14 (41.2)
*** N/A***	4 (11.8)
***CRT–* n *(%)***	23 (26.4)
***CRT timing* (% of patients treated with CRT, n = 23) *–* n *(%)***	
*** Concurrent—*n *(%)***	18 (78.3)
*** Sequential—*n *(%)***	5 (21.7)
***Trimodal treatment (CRT + surgery) –* n *(%)***	21 (24.1)

CRT: Chemoradiotherapy; NA: not available.

### Management of metastatic disease

Patients developing metastatic disease at any point during the disease course (*N = *187; Table 3), 109 (58.3%) received systemic therapy, while 78 (41.7%) received best supportive care alone, mainly due to poor performance status.

**Table 3:** Treatment distribution in metastatic disease.

Characteristic	*N = *187
***Treatment distribution by line of therapy***	
*** 1st line therapy—*n *(%)***	
*** BSC alone***	78 (41.7)
*** Chemotherapy***	47 (25.1)
*** TT***	19 (10.2)
*** TKIs monotherapy***	19 (10.2)
*** IO monotherapy***	14 (7.4)
*** TKIs + IO combination***	10 (5.3)
*** 2nd line treatment—*n *(%)***	
*** None (deceased due to progression after 1st line therapy)***	112 (59.9)
*** BSC alone***	43 (23)
*** Chemotherapy***	16 (8.6)
*** IO monotherapy***	8 (4.3)
*** TKIs + IO combination***	4 (2.1)
*** TT***	3 (1.6)
*** TKIs monotherapy***	1 (0.5)
***Treatment distribution by type of therapy (any line)***	
***Chemotherapy—*n *(%)***	59 (31.6)
*** TT—n (%)***	22 (11.7)
*** Dabrafenib + Trametinib***	20 (10.7)
*** *ALK *inhibitor***	1 (0.5)
*** FGFR inhibitor***	1 (0.5)
*** IO—*n *(%)***	41 (21.9)
*** Alone***	26 (13.9)
*** In combination with TKIs***	15 (8)
*** TKIs—*n *(%)***	43 (23)
*** Monotherapy***	28 (15)
*** In combination with IO***	15 (8)

BSC: best supportive care; IO: immunotherapy; TKI: tyrosine kinase inhibitor; TT: targeted therapy.

In the first-line setting, the most frequently administered therapy was chemotherapy, used in 47 patients (25.1%), followed by TTs and TKI monotherapy (19 patients each; 10.2%), IO (14 patients; 7.4%) and IO-TKIs combination (10 patients; 5.3%). The most common chemotherapy regimen was the combination of carboplatin and paclitaxel (used in 31 patients, 16.6%). Regarding TTs, the combination of dabrafenib and trametinib for *BRAF* V600E-mutated ATC was the most frequent regimen (17 patients; 9.1%). In addition, one patient with an *ALK* rearrangement received Ceritinib as first-line therapy. Among TKIs, lenvatinib was the most used agent (17 patients; 9.1%).

Second-line treatment was administered in 37 patients (19.8%), chemotherapy being the most frequent modality (16 patients, 8.6%), followed by IO (8 patients 4.3%) and combinations of TKIs-IO (4 patients, 2.1%). Of note, one patient with a FGFR1 amplification received TT with a FGFR1-4 inhibitor within a clinical trial as second line treatment. Only 10 patients (5.3%) received third-line treatment.

Across all lines of therapy, 43 (23%) patients received TKIs, 41 (21.9%) received IO (13.9% as monotherapy and 8% in combination with TKIs), and 22 (11.8%) received TTs. Lenvatinib was also the most frequently used TKIs across lines, accounting for 89.3% of all TKIs-based regimens. Of the patients receiving IO, 54% received single ICI agent (anti-PD-1 or PD-L1) and 46% combined ICI blockage (anti-CTLA-4 and anti-PD-L1). Of patients with targetable alterations (*n = *39), 53.8% received TTs, which mainly consisted of dabrafenib-trametinib in patients with *BRAF* V600E-mutated ATC (representing 95.2% of all TTs).

### Overall survival and prognostic associations

At the data cutoff, after a median follow-up of 4.6 months (IQR 1.2–9.7 months), 31 patients (14.5%) were alive and 174 (81.3%) had died due to ATC progression. The median OS of the cohort was 4.53 months (95%CI: 3.57–5.50). OS rates at 3, 6, 9, and 12 months were 58.9%, 39.3%, 29.4%, and 21.5%, respectively.

Significant differences were observed in OS across disease stages: 6.6 months in stage IVa (*n = *17); 5.1 months in stage IVb (*n = *69) and 4.2 months in stage IVc (*n = *115) (*p = *0.044). The presence of lung metastases was associated with shorter OS (patients with lung metastasis [*n = *79]: OS 4.3 months vs. without lung metastases [*n = *89]: OS 5.6; *p = *0.003). No prognostic associations were found with other less common metastatic locations. In this cohort, access to molecular studies (*n = *147) was associated with significantly improved OS (5.7 months vs. 1.5 months in those without them [*n = *33]; *p <* 0.001). The presence of *BRAF* V600E mutation was independently associated with better OS (9.7 months [*n = *37] vs. 4.1 months in wild type [*n = *110]; *p = *0.001), while *TERT*p mutations correlated with worse OS (5.5 months [*n = *12] vs. 11.7 months in wild type [*n = *42]; *p = *0.047). No significant differences were observed based on *TP53* status (OS in patients with ATC and TP53 mutation: 7.1 months vs. 8.2 months in wild-type ATC; *p = *0.84) or *RAS* status (OS in *RAS*-mutated ATC [*n = *20]: 4.37 months vs. 7.7 months in wild-type [*n = *80]; *p = *0.39). The expression of PAX8 by IHC was associated with longer survival (19.6 months [*n = *22] vs. 5.6 months in PAX8 negative [*n = *52]; *p = *0.034). Combining these molecular features, we identified that patients that were *TP53* and *TERT*p wild-type and with conserved PAX8 by IHQ (*TP53*-/*TERT*-/PAX8+) were a subgroup of significant better prognosis (OS 15.6 months [*n = *13]) compared to those that had ≥1 of these alterations (OS 5.8 months [*n = *80]; HR 0.38; [95%CI: 0.18–0.78]; *p = *0.009).

**Figure 1:**
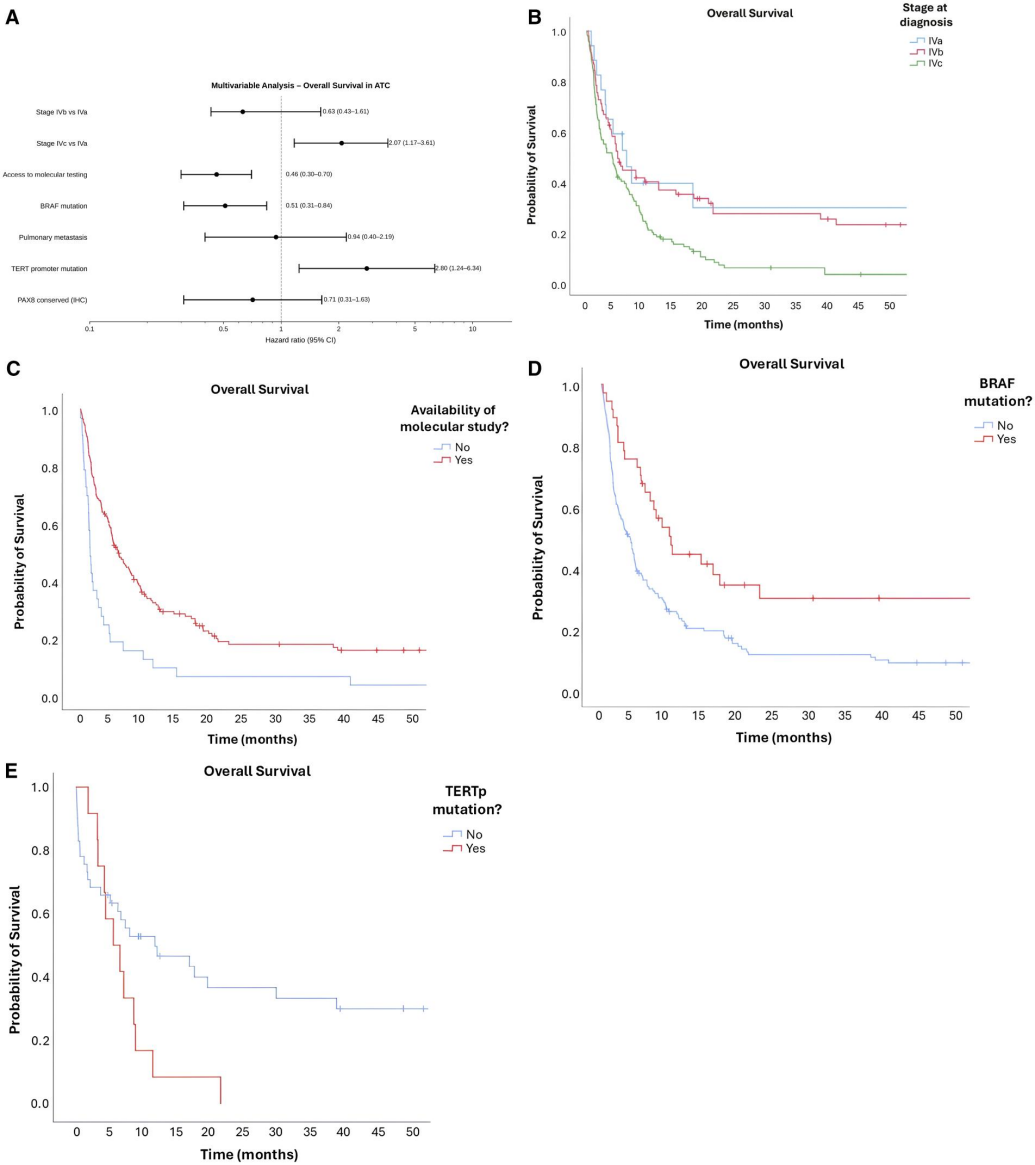
Baseline prognostic factors in ATC. Multivariable analysis of factors that were individually associated with significant Overall Survival differences (A). Kaplan-Meier Overall Survival curves stratified according to disease stage (B), access to molecular testing (C) *BRAF* status (D) and *TERTp* status (E). **Legend**: CI: confidence interval; IHC: immunohistochemistry; TERTp: TERT promoter.

However, in a multivariate analysis model (Figure 1A), the only factors that retained an independent prognostic association were disease stage (Figure 1B; HR for stage IVc vs. IVa: 2.07; *p = *0.001), access to molecular studies (Figure 1C; HR: 0.46; *p = *0.001), *BRAF* V600E mutation (Figure 1D; HR: 0.51; *p = *0.008) and *TERT*p mutation (Figure 1E; HR 2.80; *p = *0.014).

#### Locoregional disease

In stage IVa/IVb disease, local surgery was associated to improved OS outcomes (18.7 months [*n = *27] vs. 3.0 months[*n = *51]); HR 0.54 [IC95%: 0.31–0.93]; *p = *0.026), with a greater in patients with negative surgical margins (R0 resection) (49.1 months if R0 resection [*n = *8] vs. 18.7 months [*n = *19]; HR 0.34 [IC95%: 0.11–1.0]; *p = *0.050). Similarly, CRT was also associated to better OS (21.7 months [*n = *23] vs. 4.1 months in those without CRT [*n = *40]; HR 0.39; [IC95%: 0.22–0.70]; *p = *0.001).

#### Metastatic disease

Even in patients with stage IVc disease, local treatment showed prognostic relevance. Surgical resection of the primary tumor was associated with improved OS (10.6 months [*n = *23] vs. 2.7 months [*n = *75]; HR 0.34 [IC95%: 0.20–0.56]; *p <* 0.001), and a nonsignificant trend was observed for CRT (8.1 months [*n = *13] vs. 3.3 months [*n = *86]; *p = *0.36).

**Figure 2:**
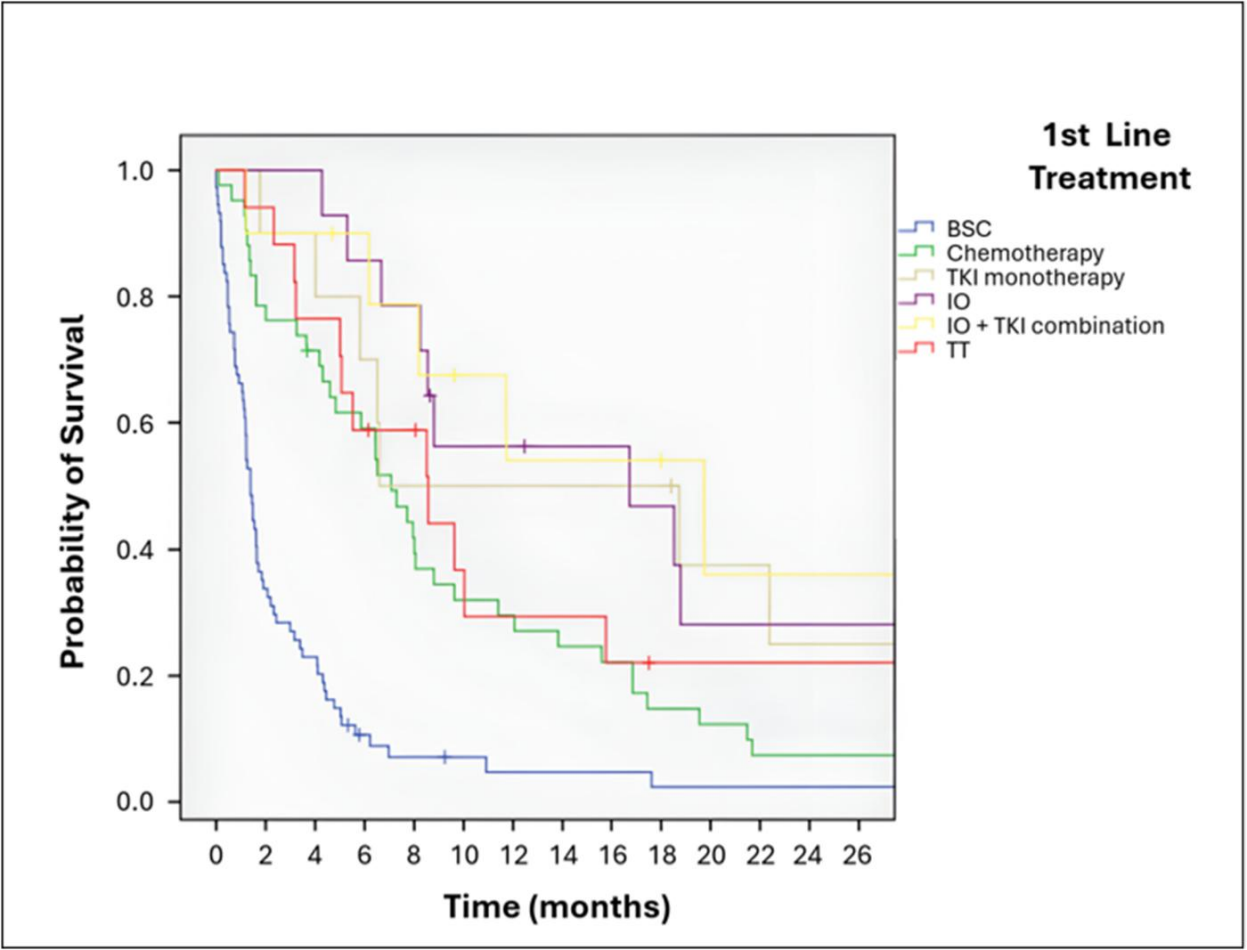
Kaplan-Meier Overall Survival curves stratified by first-line treatment. **Legend**: BSC: best supportive care; IO: immunotherapy; TKI: tyrosine kinase inhibitor; TT: targeted therapy.

In the first-line setting (Table 4 and Figure 2), the use of IO monotherapy was associated with longer OS (16.7 vs. 4.6 months; HR 0.4; *p = *0.017), as was the combination of IO with TKIs (19.8 vs. 4.6 months; HR 0.32; *p = *0.009). Conversely, best supportive care only was associated with shorter OS compared patients receiving systemic treatment (1.4 vs. 8.5 months; HR 4.17; *p <* 0.001). TTs (8.5 vs. 4.6 months; *p = *0.081) showed a non-significant trend toward improved OS.

**Table 4:** OS stratified by first line treatment.

1st line treatment	n	OS (95%CI)	HR (95%CI)	*p* Value
***BSC***	*n = *78	1.4 months (1.1–1.7)	4.17 (2.92–5.94)	<0.001
***TKI monotherapy***	*n = *19	6.6 months (5.9–7.3)	0.49 (0.23–1.01)	0.05
***Chemotherapy***	*n = *47	7.3 months (5.4–9.2)	0.84 (0.59–1.22)	0.36
***TT***	*n = *19	9.7 months (6.9–12.6)	0.59 (0.33–1.07)	0.08
***IO***	*n = *14	16.7 months (2.4–31.1)	0.48 (0.26–0.89)	0.02
***IO + TKI combination***	*n = *10	19.8 months (6.3–33.2)	0.32 (0.13–0.79)	0.01

BSC: best supportive care; IO: immunotherapy;. OS: overall survival; TKI: tyrosine kinase inhibitor; TT: targeted therapy.

**Figure 3:**
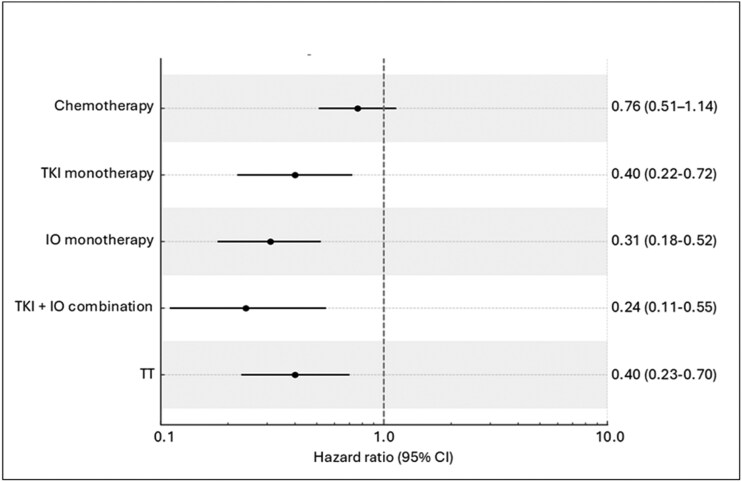
Multivariable analysis of Overall Survival by treatment across all lines. **Legend**: CI: confidence interval; IO: immunotherapy; TKI: tyrosine kinase inhibitor; TT: targeted therapy.

A multivariable analysis (Figure 3) showed that, irrespective of treatment line, patients receiving IO monotherapy (OS 16.8 months [*n = *26] vs. 3.5 months in those without IO [*n = *128]; HR 0.31; *p <* 0.001), TKI monotherapy (13.8 months [*n = *28] vs. 4.2 months in those without TKI [*n = *126]; HR 0.40; *p = *0.002), or IO–TKI combinations (11.7 months [*n = *15] vs. 4.3 months in those without IO-TKI[*n = *139]; HR 0.24; *p = *0.001) had significantly longer OS compared with those who did not receive each respective treatment. Among patients treated with TKIs or IO, no molecular subgroup (*BRAF*, *RAS*, *TP53*, or *TERT*p mutations) derived significantly different benefit. Treatment with TTs in any line was also associated with improved survival (OS 9.6 months [*n = *22] vs. 4.1 months [*n = *132]; HR 0.40; *p = *0.001). In contrast, chemotherapy was not associated to a statistically significant OS improvement (8.0 months [*n = *56] vs. 3.2 months [*n = *123]; *p = *0.096).

### BRAF-mutated ATC

Focusing on patients with *BRAF* V600E mutation (*n = *37), their baseline characteristics were overall comparable to those of *BRAF* wild-type tumors: median age 71.3 years; 56.8% females; 35.1% with locorregional disease (stage IVa/b) and 64.9% metastatic disease (stage IVc). Notably, PI3KCA mutations were more frequent in the *BRAF*-mutated ATC compared to patients with wild-type disease (20% vs. 0%; *p = *0.020), and *BRAF* and *RAS* mutations were mutually exclusive.

Management of locorregional disease in this subgroup did not differ significantly from patients *BRAF* wild-type ATC, with comparable rates of surgery (surgery rates in all stages: 31.4% in *BRAF* mutated vs. 31.4% in *BRAF* wild-type), R0 resections (R0 rates in operated patients: 27.3% in *BRAF* mutated vs. 62.9% in *BRAF* wild-type) and CRT (CRT rates in all stages: 15.6% in *BRAF* mutated vs. 24% in *BRAF* wild-type) (*p* > 0.05 for all comparisons). Conversely, systemic therapy patterns differed between patients with *BRAF* mutated and wild-type ATC (Supplemental Table 2). Patients with BRAF V600E mutation were more likely to of receiving TTs (58.8% vs. 1.9%; *p <* 0.001) and a lower likelihood of receiving chemotherapy (13.5% vs. 37.9% *p = *0.006). All patients with *BRAF*-mutated ATC that received TTs were treated with Dabrafenib-Trametinib, which was used as first-line treatment in 50% of patients.

At the data cut-off, 35.2% of patients with *BRAF*-mutated ATC were alive and 67.6% had died due to ATC progression. As previously described, the population *BRAF*-mutated ATC had a significantly better OS compared to wild-type ATC (9.7 vs. 4.1 months). Treatment with dabrafenib-trametinib in this population (*n = *20) had a median PFS of 5.8 months and an OS of 10.2 months, which was numerically higher than the OS of patients with *BRAF*-mutated ATC without targeted treatment (*n = *17; OS 5.6 months; *p = *0.24).

## Discussion

### Results of our study

The REGETNE-Thyroid cohort (*N = *214) represents, to our knowledge, one of the largest European ATC cohorts published to date. A notable strength is the high rate of molecular testing (69%), considering the challenges of performing molecular studies in such an aggressive disease. The molecular results were consistent with previous ATC reports, with *TP53* and *TERT* promoter mutations being most common.^3–6^ The prevalence of *BRAF* mutation (20%) was lower than in American cohorts (≈40%) but consistent with other European and Asian data, such as the ENDOCAN-TUTHYREF French cohort.^5^^,^^24–29^

Median OS was 4.5 months, in line with the poor prognosis historically reported in ATC.^2^^,^^30–34^ Independent baseline prognostic factors included access to molecular testing and *BRAF* mutation, both associated with improved survival, and *TERT* promoter mutation, associated with worse outcomes. These findings underscore the importance of molecular characterization, both for its prognostic and therapeutic implications.

Locoregional management also impacted outcomes. Surgery (especially if R0 resection was achieved) and CRT were both associated with longer survival in stage IVa/b disease. Importantly, local surgery also improved survival in patients with metastatic disease, with a similar, non-significand trend for CRT. This highlights the role of local control even in metastatic stages, as local complications such as airway obstruction or hemorrhage remain a leading cause of death in ATC.^17^^,^^18^^,^^26^

Systemic therapy was administered to 58% of patients with metastatic disease, while the remainder received supportive care only, highlighting the rapid clinical deterioration that precludes treatment in many cases. A total of 59% of patients with *BRAF*-mutated ATC received TTs, a noteworthy proportion given the challenges of molecular testing turnaround, and restricted drug access in real-world settings.

Treatment with IO (OS 16.8 vs. 3.5 months; HR 0.31), IO+TKI combinations (OS 11.7 vs. 4.3 months; HR 0.24), TKI monotherapy (OS 13.8 vs. 4.2 months; HR 0.40), and TTs (OS 9.6 vs. 4.5 months; HR 0.40) each conferred significant survival benefit compared with patients not receiving these therapies. Outcomes with IO and IO + TKI combinations are comparable to those reported in prior ATC trials (e.g., Durvalumab + Tremelimumab in the DUTHY trial: OS 13.8 months; Lenvatinib + Pembrolizumab in the ATLEP trial: OS 10.3 months),^20^^,^^22^ while the survival benefit observed with TKI monotherapy is substantially higher than in prior studies (e.g., a phase II trial and a meta-analysis both reported an OS of ∼3.2 months).^21^^,^^35^ Regarding TTs, the benefit observed in our cohort was largely driven by dabrafenib + trametinib, which represented >95% of TTs. Particularly in *BRAF*-mutated ATC, dabrafenib + trametinib had a PFS and OS of 5.8 and 10.2 months, respectively. These results are slightly inferior to the ROAR trial (PFS 6.7; OS 14.7 months),^10^ which may be due to the much lower use of local surgery/CRT in this subpopulation in our cohort (32% vs. >80% in the ROAR trial), again emphasizing its prognostic impact even in metastatic settings. Although these results should be interpreted with caution due to the retrospective design and limited sample size, they reinforce the clinical relevance of IO, TKIs, their combinations, and TTs as therapeutic strategies in ATC. Therefore, facilitating access to these treatments for eligible patients is essential. Although chemotherapy remained the most frequently administered treatment, its survival benefit was limited compared with other systemic therapies. This disparity highlights the persistent gap between clinical trial evidence and real-world practice, where access to more novel therapies is often limited.

Finally, two points warrant consideration. First, given the well-established association of *BRAF* V600E with aggressive tumor biology across cancer types,^36–39^ the improved survival observed in patients with *BRAF*-mutated ATC in our study is unlikely to reflect the mutation itself but rather the efficacy of TTs in this population. Second, the association between access to molecular testing and improved OS may be partly explained by selection bias, as patients with fulminant disease often deteriorate before testing can be performed. Nonetheless, the sustained separation of Kaplan–Meier curves (Figure 1B) over time suggests that other factors, such as the benefit of molecularly directed therapies, also contributed to the observed survival advantage.

### Comparison with the ENDOCAN-TUTHYREF cohort

The ENDOCAN-TUTHYREF is another European ATC cohort (*n = *360; France, 2010–2020).^25^^,^^26^ Compared to our cohort, features were broadly similar, including age, sex, and stage at diagnosis. Molecular testing was more frequent in our cohort (69% vs. 54%), with similar mutation rates for *TP53*, *BRAF*, and *RAS*.

Local management differed, with surgery being more common in our cohort (32.7% vs. 15.9%), but CRT more frequent in the French cohort (37.8% vs. 16.8%). Rates of systemic therapy in all stages were similar (∼70%), though in the metastatic setting, supportive care was more frequent in our series (41.7% vs. 19%). IO and TTs use were higher in our cohort (IO: 21.9% vs. 5%; TT: 53.8% vs. 39.5% of patients with actionable alterations), particularly in first line.

Across both cohorts, median OS was comparable (4.5 months in REGETNE-Thyroid vs. 6.5 months in ENDOCAN-TUTHYREF), with consistent benefit from dabrafenib + trametinib use in *BRAF*-mutated ATC (REGETNE-Thyroid: PFS 5.8 months and OS 10.2 months; ENDOCAN-TUTHYREF: PFS 6.4 months and OS 14.2 months) and from IO (OS of 16.8 months in the REGETNE-Thyroid vs. 20.1 months in ENDOCAN-TUTHYREF).

### Limitations of the study

This study has several limitations. First, its retrospective and observational nature introduces inherent biases (e.g., selection, information, and recall biases), including the lack of centralized tumor samples review to confirm ATC, and potential unmeasured confounding factors may have influenced the results despite multivariable adjustments. Second, there is heterogeneity in the molecular techniques used across participating centers, according to local standards and availability. Third, although this is one of the largest European ATC cohorts published to date, the rarity of ATC limits statistical power, particularly in subgroup analyses. However, the rarity of ATC precludes having larger sample size. In fact, the REGETNE-Thyroid database has been a great multicentric effort. Lastly, the aggressive clinical course of ATC may have led to a selection bias, as patients with fulminant disease often could not undergo molecular testing or receive systemic treatment. Thus, patients with available molecular likely reflect a subset with more indolent disease.

## Conclusions

The REGETNE-Thyroid cohort represents one of the largest real-world series of ATC published to date. Molecular profiling was associated with improved outcomes, likely driven by access to targeted therapies, and enabled the identification of prognostic subgroups. Local treatments were associated with improved survival even in metastatic settings, highlighting their importance across all stages. Although chemotherapy was the most commonly used treatment, only TTs, IO, TKIs, and IO + TKI combinations were associated with survival benefits. These findings highlight the need to ensure timely access to such treatments and reinforce the importance of multidisciplinary, biomarker-guided treatment decisions.

## Supplementary Material

oyag158_Supplementary_Data

## Data Availability

The data underlying this study were obtained from the REGETNE-Thyroid Network and contain sensitive patient-level clinical information. For ethical and legal reasons, the data are not publicly available. De-identified data may be shared upon reasonable request to the corresponding author, subject to approval by the participating centers and relevant ethics committees.
