# Supplementary material for: Real-world outcomes and prognostic factors in anaplastic thyroid cancer: evidence from the REGETNE-Thyroid cohort
Source: Oncologist. 2026 May 21;31(7):oyag158. doi: 10.1093/oncolo/oyag158 (PMC13243708; doi:10.1093/oncolo/oyag158)
Supplement: oyag158_Supplementary_Data [file oyag158_supplementary_data.zip › Supplemental Table 2.docx]

**Supplemental Table 2: Treatment distribution in metastatic *BRAF*-mutated ATC.**

| **Characteristic** | **N=34** |
| --- | --- |
| **Treatment distribution by line of therapy** | |
| **1^st^ line therapy** – n (%) | |
| BSC alone | 7 (20.6) |
| TT | 17 (50) |
| Chemotherapy | 3 (8.8) |
| IO monotherapy | 3 (8.8) |
| TKIs + IO combination | 3 (8.8) |
| TKIs | 1 (2.9) |
| **2^nd^ line treatment** – n (%) | |
| None (deceased due to progression after 1st line therapy) | 19 (55.9) |
| BSC alone | 9 (26.5) |
| TT | 3 (8.8) |
| Chemotherapy | 2 (5.8) |
| TKIs monotherapy | 1 (2.9) |
| IO monotherapy | 0 (0) |
| TKIs + IO combination | 0 (0) |
| **Treatment distribution by type of therapy (any line)** | |
| **Chemotherapy** – n (%) | 5 (14.7) |
| **TT** *(all treated with Dabrafenib + Trametinib)*– n (%) | 20 (58.8) |
| **IO** – n (%) | 6 (17.6) |
| Alone | 4 (11.7) |
| In combination with TKIs | 2 (5.9) |
| **TKIs – n (%)** | 5 (14.7) |
| Monotherapy | 2 (5.9) |
| In combination with IO | 3 (8.8) |

*BSC: best supportive care; IO: immunotherapy; TKI: tyrosine kinase inhibitor;TT: targeted therapy.*
