# Supplementary material for: Real-world outcomes and prognostic factors in anaplastic thyroid cancer: evidence from the REGETNE-Thyroid cohort
Source: Oncologist. 2026 May 21;31(7):oyag158. doi: 10.1093/oncolo/oyag158 (PMC13243708; doi:10.1093/oncolo/oyag158)
Supplement: oyag158_Supplementary_Data [file oyag158_supplementary_data.zip › Supplemental Table 1.docx]

**Supplemental Table 1: Clinical trials exploring immunotherapy and/or TKIs in patients with ATC**

| **Clinical trials** | **Investigational drug(s)** | **ATC cohort size** | | **ORR** | | **PFS** | | **OS** |
| --- | --- | --- | --- | --- | --- | --- | --- | --- |
| **NCT02404441** | **Spartalizumab** | N=42 | | 19% | | 1.7 months | | 5.9 months |
| **DUTHY (GETNE T1812)**  **(EudraCT 2018-001066-42)** | **Durvalumab + Tremelimumab** | N=12 | | 33% | | 4 months | | 13.8 months |
| **ATLEP (EudraCT 2017-004570-3)** | **Lenvatinib + Pembrolizumab** | N=27 | | 51.9% | | 9.5 months | | 10.3 months |
| **CABATEN/ GETNE-T1914 (NCT04400474)** | **Cabozantinib + Atezolizumab** | N=14 | | 21.4% | | 8.4 months | | N/A |
| **NCT03181100 (cohort 1, patients *BRAF*V600E mutation)** | **Atezolizumab + Vemurafenib + Cobimetinib** | N=19 | | 50% | | 13.9 months | | 43 months |
| **NCT03181100 (cohort 2, pacients with *RAS*/NF1 mutations)** | **Atezolizumab + Cobimetinib** | N=21 | | 14% | | 4.8 months | | 13.9 months |
| **NCT03181100 (cohort 3, pacients without *BRAF*/*RAS*/NF1 mutations)** | Atezolizumab +  Bevacizumab | N=3 | 30% | | 1.3 months | | 6.8 months | |
| **NCT03181100 (cohort 3, pacients without *BRAF*/*RAS*/NF1 mutations)** | Atezolizumab + Paclitaxel | N=9 | 11% | | 2.7 months | | 4.4 months | |

*ATC: anaplastic thyroid cancer; IO: immunotherapy; ORR: overall response rate; OS: overall survival; PFS: progression free survival; TKIs: tyrosine kinase inhibitors.*
